# Association of DGAT1 With Cattle, Buffalo, Goat, and Sheep Milk and Meat Production Traits

**DOI:** 10.3389/fvets.2021.712470

**Published:** 2021-08-16

**Authors:** Muhammad Zahoor Khan, Yulin Ma, Jiaying Ma, Jianxin Xiao, Yue Liu, Shuai Liu, Adnan Khan, Ibrar Muhammad Khan, Zhijun Cao

**Affiliations:** ^1^State Key Laboratory of Animal Nutrition, Beijing Engineering Technology Research Center of Raw Milk Quality and Safety Control, College of Animal Science and Technology, China Agricultural University, Beijing, China; ^2^Faculty of Veterinary and Animal Sciences, Gomal University, Dera Ismail Khan, Pakistan; ^3^Shenzhen Branch, Guangdong Laboratory for Lingnan Modern Agriculture, Genome Analysis Laboratory of the Ministry of Agriculture, Agricultural Genomics Institute at Shenzhen, Chinese Academy of Agricultural Sciences, Shenzhen, China; ^4^Anhui Provincial Laboratory of Local Livestock and Poultry Genetical Resource Conservation and Breeding, College of Animal Science and Technology, Anhui Agricultural University, Hefei, China

**Keywords:** DGAT1, polymorphisms, milk production, meat production, genetic marker

## Abstract

Milk fatty acids are essential for many dairy product productions, while intramuscular fat (IMF) is associated with the quality of meat. The triacylglycerols (TAGs) are the major components of IMF and milk fat. Therefore, understanding the polymorphisms and genes linked to fat synthesis is important for animal production. Identifying quantitative trait loci (QTLs) and genes associated with milk and meat production traits has been the objective of various mapping studies in the last decade. Consistently, the QTLs on chromosomes 14, 15, and 9 have been found to be associated with milk and meat production traits in cattle, goat, and buffalo and sheep, respectively. Diacylglycerol *O*-acyltransferase 1 (DGAT1) gene has been reported on chromosomes 14, 15, and 9 in cattle, goat, and buffalo and sheep, respectively. Being a key role in fat metabolism and TAG synthesis, the DGAT1 has obtained considerable attention especially in animal milk production. In addition to milk production, DGAT1 has also been a subject of interest in animal meat production. Several polymorphisms have been documented in DGAT1 in various animal species including cattle, buffalo, goat, and sheep for their association with milk production traits. In addition, the DGAT1 has also been studied for their role in meat production traits in cattle, sheep, and goat. However, very limited studies have been conducted in cattle for association of DGAT1 with meat production traits in cattle. Moreover, not a single study reported the association of DGAT1 with meat production traits in buffalo; thus, further studies are warranted to fulfill this huge gap. Keeping in view the important role of DGAT1 in animal production, the current review article was designed to highlight the major development and new insights on DGAT1 effect on milk and meat production traits in cattle, buffalo, sheep, and goat. Moreover, we have also highlighted the possible future contributions of DGAT1 for the studied species.

## Introduction

Milk production traits have fundamental importance in livestock production and the related economy (1, 2). The milk fatty acids have shown an essential role in cheese production, and it has been found that milk fat includes about 98% triglycerides (TGs) (3). In addition to milk production, scientists are also taking an interest in the quality of meat. The trends for meat quality instead of meat yield are gradually changing in many countries (4, 5). In order to improve productivity, the animal with better quality traits such as milk production, growth, meat, and carcass quality have been selected and used in the breeding program in the animal industry.

Genetic factors can influence milk fat composition, and its genetic variation has been reported in previous studies (6, 7). Consistently, it has been documented that the carcass and meat quality traits are under the control of several genes (4). Selection aimed at increasing the frequency of alleles with a positive effect on a given trait was initiated by geneticists (8). In general, identifying and validating genetic markers for milk production traits are the initial and crucial steps to establish a marker-assisted selection (MAS) system. Thus, the increasing productive performance through genetic selection is a common goal for many animal breeding programs worldwide (9–11).

Several studies have targeted the bovine quantitative trait loci (QTLs) on chromosome 14 for their association with milk production traits (12–20). Acyl CoA:diacylglycerol acyltransferase (DGAT1) gene at chromosome 14 has been documented as a candidate marker for the QTLs associated with milk production traits through cattle genome and linkage mapping studies (21, 22). Besides DGAT1 association with milk production traits, many studies have documented that the genes on bovine chromosome 14 regulate many economic traits including meat production in beef cattle (23–26). Urbinati et al. explored several genes on chromosome 14 that had significantly regulated metabolism, melanin biosynthesis (pigmentation), bone development, and meat production in dairy cattle. They have documented the pleiotropic ability of DGAT1 for both influencing meat and milk quality in dairy cattle (27). Later on, DGAT1 was reported as a production-associated gene in several animals including buffalo, sheep, and goat. Keeping in view the importance of DGA*T1* gene in animal production, the current review was designed to highlight the possible research development on DGAT1 role in cattle, buffalo, sheep, and goat milk and meat production trait regulation.

## Short Description of DGAT1 in Cattle, Buffalo, Sheep, and Goat

Diacylglycerol *O*-acyltransferase 1 (DGAT1), Ensembl ID (Ensembl: ENSBTAG00000026356) was identified as one underlying QTL for milk production traits located on the centromeric region of the bovine chromosome 14 having 17 exons with 14,117 base pair (bp) (21, 22). *DGAT1* gene (Gene ID: 102390126) in buffalo located on chromosome 15 has a size of 10,733 bp distributed in 19 exons (28). The expression of DGAT1 has been documented in the small intestine, liver, adipose tissue, and mammary gland (29, 30). In sheep, *DGAT1* (Gene ID: 100126245) is located on chromosome 9 and consisted of 17 exons (31), while in goat, *DGAT1* gene (Gene ID: 100861225) is located on chromosome 14 with 18 exons (https://www.ncbi.nlm.nih.gov/gene/100861225).

## DGAT1 Role in Mammary Gland Development and Synthesis of Triacylglycerol

**Figure 1:**
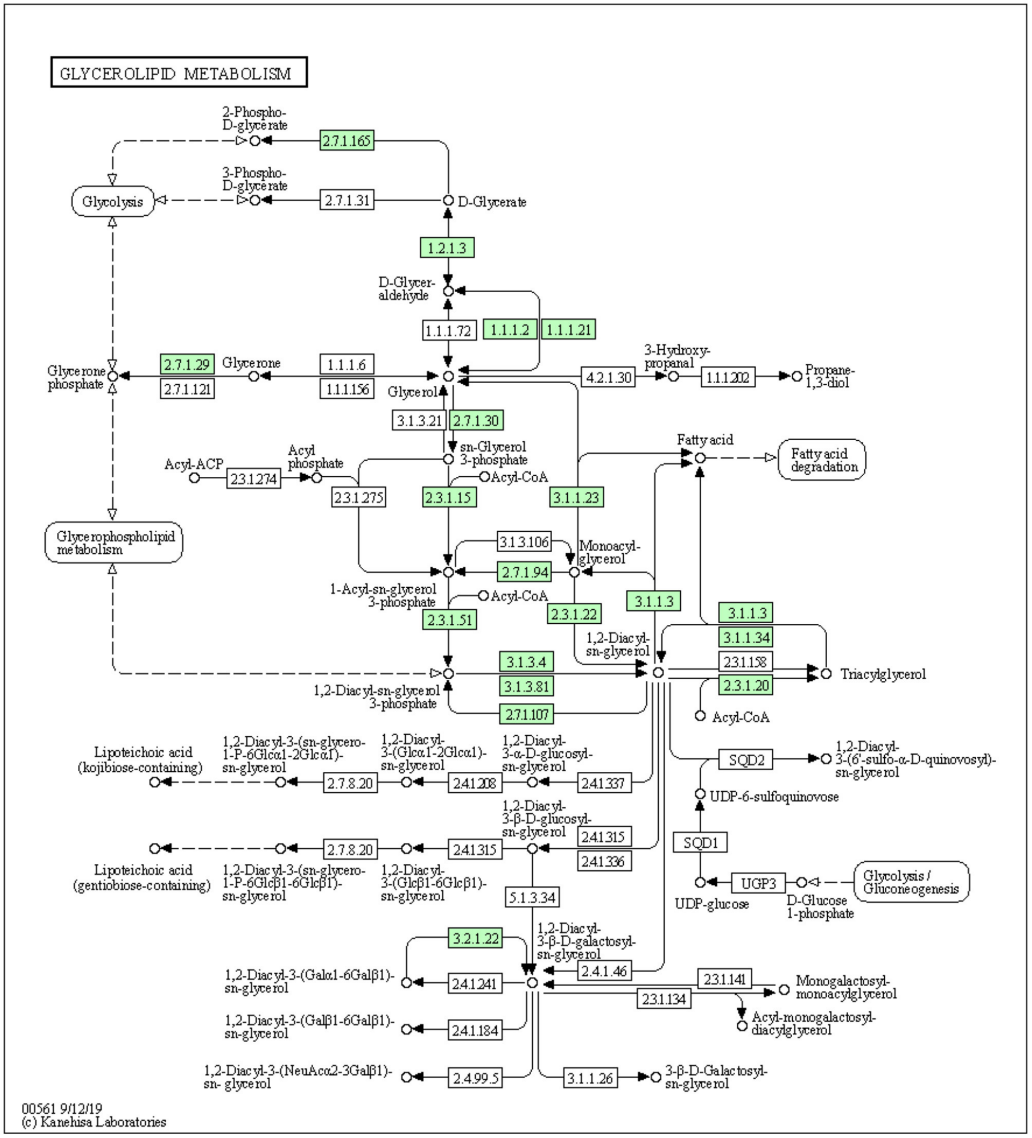
The synthesis of triacylglycerol by DGAT1 using glycerol-3-phosphate pathway (https://david.ncifcrf.gov/kegg.jsp?path=bta00561$Glycerolipid%20metabolism&termId=550009448&source=kegg).

The development of the mammary gland is a complex process that is necessary for normal lactation. The interactions between the mammary epithelium and surrounding stroma are crucial for normal mammary gland development. Furthermore, adipocytes are the most abundant cells in the stroma. Stromal adipocytes synthesize and store large amounts of TGs, which may serve as reservoirs of substrates for milk production by the mammary epithelium. During this process, adipocyte TGs must be hydrolyzed and the fatty acids transferred to the epithelial cells for re-esterification. TG synthesis is catalyzed by diacylglycerol *O*-acyltransferase 1 (DGAT1) enzymes, which covalently join diacylglycerol with fatty acyl CoA (Figure 1) (32). A study has identified genes encoding two mammalian DGAT enzymes, DGAT1 and DGAT2 (33). *DGAT1* gene is expressed in nearly all tissues, including the mammary glands (34). Mice lacking DGAT1 (*Dgat1*^−/−^) have decreased TG content in tissues and cannot lactate (35). Furthermore, Cases et al. found that the mice lacking DGAT1 had faced impaired mammary gland with decreased epithelial proliferation and alveolar development. Similarly, it has been documented that the insufficient level of DGAT1 in both the stromal and epithelial tissues is associated with impaired mammary gland development (36). The knockdown of DGAT1 in bovine mammary epithelial was associated with a reduced level of TG synthesis in bovine mammary epithelial cells (BMECs) (37).

### DGAT1 Role Association With Fat Metabolism and Milk Production Traits

**Figure 2:**
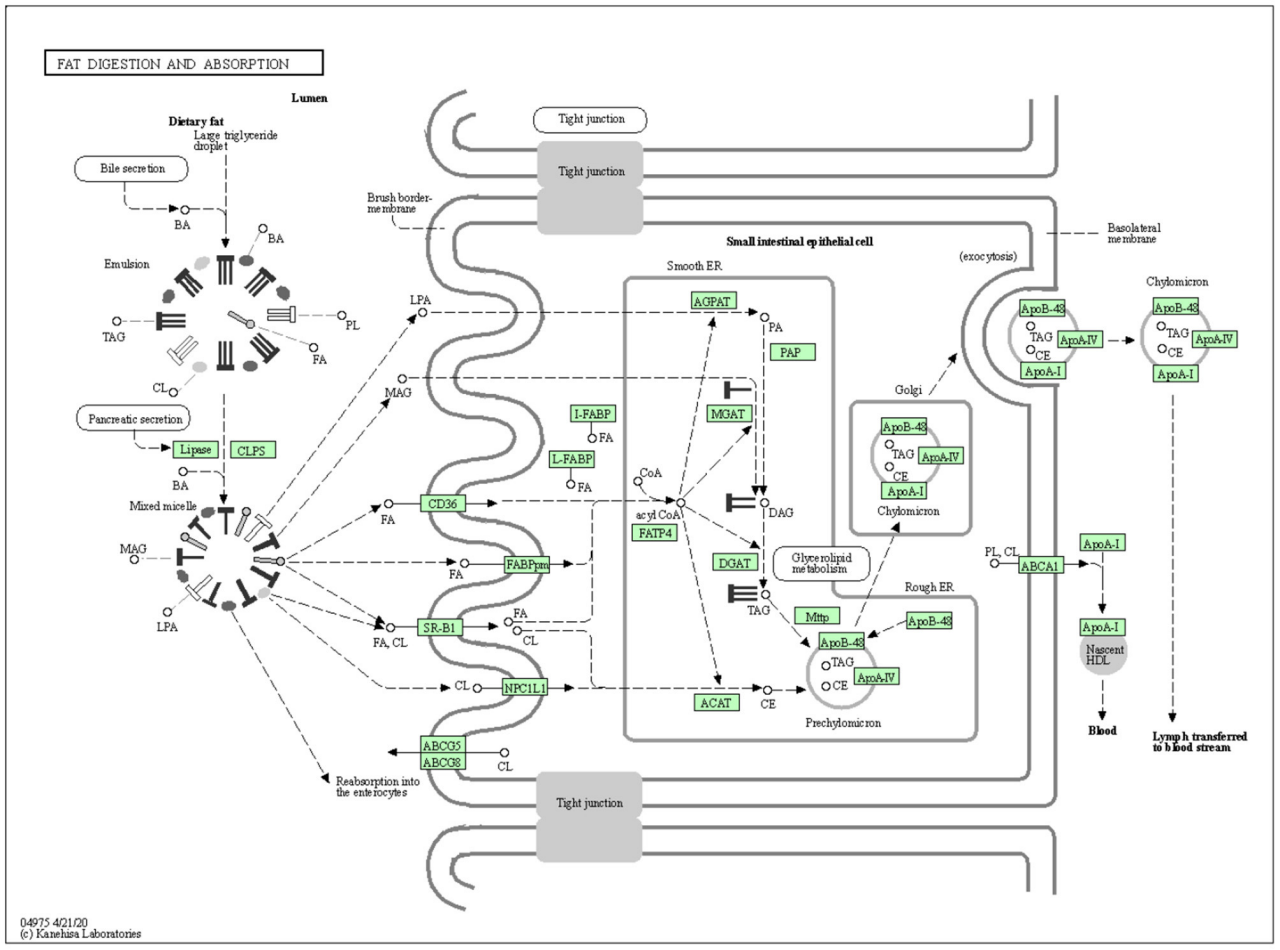
The mechanism of DGAT1 role in fat digestion and metabolism (https://david.ncifcrf.gov/kegg.jsp?path=bta04975\protect\LY1\textdollarFat%20digestion%20and%20absorption&termId=550009625&source=kegg).

The vital role of DGAT1 in fat metabolism makes them a best choice as a candidate marker in animal production. The mechanism of DGAT1 role in fat metabolism is shown in Figure 2.

Consequently, DGAT1 was documented to have a significant influence on milk production in cattle in Germany (38). Ardicli et al. documented the association of DGAT1 with milk production traits in dairy cattle and concluded that DGAT1 could be used as a genetic marker to improve the milk production traits (39, 40). Through the genome-wide association study (GWAS), it has been well established that DGAT1 is associated with milk production traits (41–43). Consistently, Jiang et al. documented through a GWAS that *DGAT1* gene is correlated with milk fat yield; however, a negative link of single-nucleotide polymorphism (SNP) (rs109421300) in DGAT1 with milk and protein yields was noticed in U.S. Holstein cattle (44). The SNP (rs109421300) has A and G alleles, while G allele was associated with antagonistic pleiotropy for positive fat yield and negative milk and protein yields in U.S. Holstein cattle. It was reported in a recent study that the variant DGAT1 K232A modulates the expression of *DGAT1* gene in the mammary gland of dairy cattle (19).

### DGAT1 K232A Association With Milk Production in Cattle

**Figure 3:**
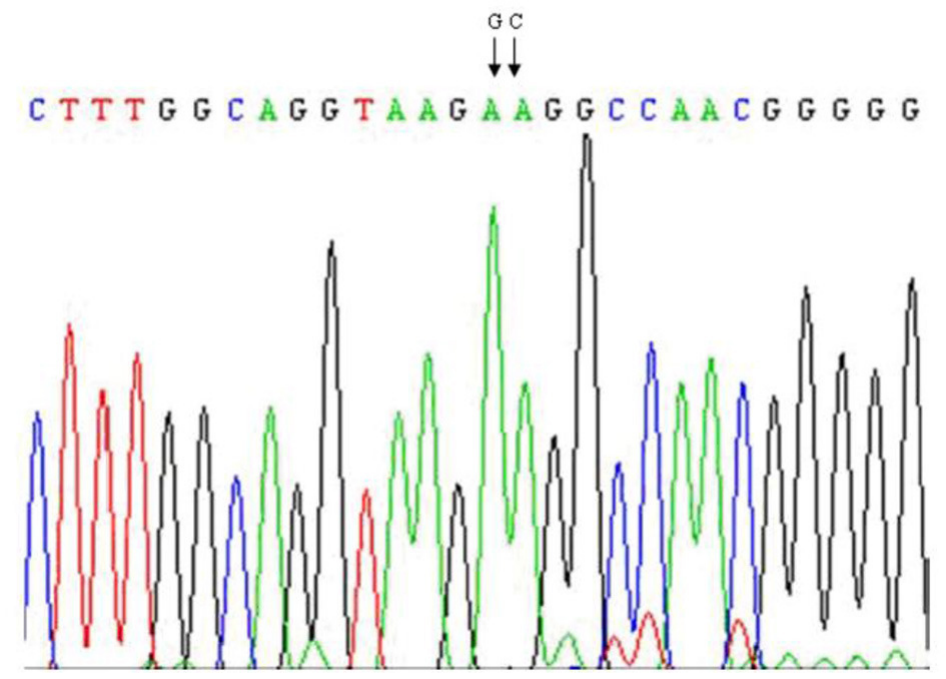
The AA → GC (K232A) mutation in exon 8 of DGAT1 in dairy cattle.

A polymorphism AA → GC exchanges and causes the substitution of amino acid 232. Lysine (K) → alanine (A) was detected on exon 8 of *DGAT1* gene of *Bos taurus:* (Figure 3) (21, 22, 45). Later on, many studies documented the significant association of DGAT1 K232A polymorphism with milk production traits in dairy cattle (45–49). The DGAT1 region analysis in six Indian cattle (Sahiwal, Rathi, Deoni, Tharparkar, Red Kandhari, and Punganur) revealed fixed DGAT1^K^ allele (Figure 3) (45). Moreover, Tania et al. documented that the desired region sequence of control cows showed heterozygous situation for already detected mutation in three cows.

The allelic frequency and the influence of the DGAT1 K232A polymorphism have been illustrated in dairy cattle populations in different countries including New Zealand (12, 19, 21, 50), Israel (13), the Netherlands (51, 52), Germany (53, 54), Poland (55, 56), China (57–59), France (60), India (45), and Sweden (47).

Furthermore, it has been well established that the DGAT1 K allele is linked to a higher fat yield, fat content, and protein content and lower milk production protein and lactose yield (42, 46, 47, 54). Few reports also studied that the increase in milk fat and protein percentages was associated with the K variant in DGAT1, while the increase in milk yield was linked to the A variant in dairy cattle (40, 47). Berry et al. documented that the K allele of DGAT1 K232A is significantly associated with higher milk fat content, protein content, and milk fat yield and lower milk and protein yield in dairy cattle in Denmark (61). Bovenhuis et al. (42) found that the changes in milk production traits during early lactation might be due to DGAT2 (33) in addition to DGAT1. Furthermore, Bovenhuis et al. showed that milk protein is directly associated with phosphate, calcium, and magnesium contents; thus, DGAT1 K232A polymorphism may change the milk mineral composition (42). In a recent study, the lactation stage interaction with genotypes (*SNP* × *lact*) was evaluated for seven milk production traits (milk yield, lactose yield, lactose content, fat yield, fat content, protein yield, and somatic cell score) in 1,800 first-parity Dutch Holstein–Friesian (20). It has been reported that DGAT1 K232A effect on milk production changes with lactation stage (20, 42). The influence of DGAT1 K232A on milk yield tends to decrease in the early lactation stage when the cows experience negative energy balance (NEB) (42); however, the effect was constant for milk fat throughout the lactation but slightly lowered in early lactation (20). The main reasons documented for these changes were NEB (42) and effects of pregnancy because repartitioning of nutrients is required between milk production and fetus development (18, 20). Furthermore, it is hypothesized that DGAT2 shows more critical role than DGAT1 in early lactation, which could be another contributory factor to the above-observed changes (42). In contrast, it was noticed by a study that DGAT1 effects increased in all the three stages of lactation day 5–95, from day 96 to 215, and from day 216 to 305 (62). Lu and Bovenhuis assumed that pregnancy in the late stage of lactation might be one reason for changes in milk production contents, especially lactose contents (18). Mao et al. evaluated the effects of the DGAT1 genotypes on milk production traits in dairy cattle; it was confirmed through an association study that dairy cows with KK genotype were significantly superior in milk fat and protein contents compared with AA and KA. In contrast, higher 305-day fat yield and 305-day protein yield were reported in cows with genotype KA compared with AA or KK genotypes (61). A recent study reported the interactions of DGAT1 K232A with milk fat and protein percentages. In addition, the lactation stage was the critical factor associated with the effect of DGAT1 polymorphism on milk fat and protein percentages. The AA genotype was linked to a reduction in milk fat percentage, while KK significantly increased milk protein percentage (49). Venbergue et al. selected 21 dairy cows and divided them into three groups based on the DGAT1 K232A genotype (eight cows each of KK and KA genotype; five cows of AA).

Furthermore, the KK genotype cows presented higher milk fat and protein contents, higher κ-casein percentage, and lower milk yield (63). Consequently, the DGAT1 AA genotype was associated with low milk fat and protein contents and higher milk yield and lactose yield than the DGAT1 KK genotype in dairy cattle (64). Consistently, it has been found that the K allele in DGAT1 is linked to higher milk fat and protein contents, while the A allele showed association with higher milk yield in dairy cattle (7, 42, 65). In contrast, Mach et al. did not find any significant effect of DGAT1 polymorphism on milk production traits in dairy cattle (66). A study proved through a gene expression experiment that the enzyme activity level of the alanine variant was lesser than that of the lysine variant (51). Interestingly, Demeter et al. explored the effect of DGAT1 polymorphism on non-return rates for insemination. Furthermore, the findings of Demeter et al. provided a roadmap to determine the associated effects of breeding to improve the composition of milk fat on reproduction, thus enabling a better assessment of breeding programs prior to implementation (52).

Few studies have reported that the effect of DGAT1 K232A also depends on the breed (53, 67), lactation stage (68, 69), and parity (41, 60). Furthermore, it has been documented that milk fat contents were not constant throughout lactation in dairy cattle (68, 69). The genetic effect of DGAT1 on milk production traits showed interaction with the lactation stage (18, 20). The possible reasons for the decreasing effects of DGAT1 on milk production traits, especially on milk protein contents during the first and second lactation, might be due to the dilution effect and *de novo* fatty acid synthesis (42). Consistently, it has been reported that in early lactation, dairy cows experienced a NEB. The dairy cows mobilize body reserves to fulfill energy deficiency utilized to maintain milk yield (41). Bionaz et al. (70) provided evidence for differential expression of DGAT1 during lactation. They showed that significant changes in DGAT1 expression only occurred in early lactation (<15days), whereas in late lactation, no significant changes in DGAT1 expression were detected. Consistently, few studies also documented that DGAT1 expression in the bovine mammary gland alters with the initiation of lactation (70–73). However, these studies noticed that the changes in DGAT1 expression were not constant throughout lactation in dairy cattle.

In addition, Lu et al. studied the effect of DGAT1 K232A polymorphism on milk metabolome and proteome obtained from two groups of cows (AA and KK genotype cows). It was found that milk from cows with KK genotypes consisted of less uridine diphosphate (UDP)-linked sugar, citrate and creatine, and more stomatin, choline, carnitine, and sphingomyelin when compared with milk obtained from cows having AA genotypes. Thus, the cows different in DGAT1 polymorphism showed variations in milk metabolome and proteome, which helps us to understand the mechanism behind the effect of DGAT1 K232A polymorphism on milk production characteristics (37).

The genetic effect of DGAT1 polymorphism on milk production is also affected by the season. Recently, a study noticed the significant effect of winter and summer on the genetic effect of DGAT1 polymorphism on milk production traits; in winter, the DGAT K232A effect was negatively associated with milk fat (74). However, neither increasing nor decreasing effect of allele DGAT K232A was reported on milk content traits with change in temperature in the Polish Holstein–Friesian breed. However, some of the milk production traits (milk protein yield and milk yield) were not constant throughout the lactation and slightly decreased with change in environmental temperature (20–28°C). The more prominent effect of DGAT K232A in Polish Holstein–Friesian was not observed because the temperature was moderate (5°C−28°C) in this study (75). Shahzad et al. noticed the low hepatic expression of DGAT1 in periparturient dairy cattle during summer compared with spring (76). Consistently, a study reported that the KK genotype of DGAT K232A polymorphism showed association with triacylglycerol (TAG) composition in milk fat in summer, while in winter, the effect among genotypes (AA cow and KK cow) was smaller for TAG composition in milk (77). Bovenhuis et al. documented the effect of DGAT1 polymorphism on milk, fat, protein yield, and fat and protein content. Furthermore, it was noticed that DGAT1 polymorphism also influenced lactose content and lactose yield (42). In addition to DGAT K232A, the DGAT1 variable number of tandem repeat (VNTR) was also found to be associated with milk fat percentage in dairy cattle. The promoter VNTR polymorphism has shown effect on specificity protein 1 (SP1) binding sites and regulate the expression of *DGAT1* gene as well (78). The research development on DGAT–K232A has also been summarized in Table 1.

**Table 1:** Research development on DGAT1 K232A polymorphism association with milk production traits in dairy cattle.

**Polymorphism**	**Production traits**	**Country**	**Breeds**	**References**
DGAT1 K232A	Milk production traits	Belgium	Holstein–Friesian	Grisart et al. (51)
DGAT1 (K232A)	Milk fatty acid traits	Benin	Borgou and White Fulani	Houaga et al. (79)
DGAT1 K232A	Milk production traits	Brazil	Nellore, Guzerat, Red Sindhi, Gyr, Holstein, and Gyr × Holstein	Lacorte et al. (80)
DGAT1 K232A	Milk fatty acids	Chile	Holstein–Friesian, Jersey, Frisón Negro, Montbeliarde, and Overo Colorado	Carvajal et al. (81)
DGAT1 K232A	Milk production traits	China	Chinese local breed	Li et al. (82)
DGAT1 K232A	Milk production traits	Czech Republic	Holstein–Friesian	Kadlecova et al. (83)
DGAT1 K232A	Milk production traits	France	French Holstein, Normande, and Montbéliarde	Gautier et al. (60)
DGAT1 K232A	Milk production traits	France	Holstein × Normande crossbred	Vanbergue et al. (63)
DGAT1 K232A	Milk production traits	Germany	Holstein–Friesian, Fleckvieh and Braunvieh	Winter et al. (22)
DGAT1 K232A	Milk fat	Germany	German Holstein	Kuhn et al. (84)
DGAT1 K232A	Milk production traits	Germany	German Holstein	Kuhn et al. (85)
DGAT1 K232A	Milk production traits	India	Sahiwal, Rathi, Deoni, Tharparkar, Red Kandhari and Punganur	Tantia et al. (45)
DGAT1 (K232A)	Milk production traits	Ireland	Holstein–Friesian	Berry et al. (61)
DGAT1 K232A	Milk fat	Israel	Dutch Holstein–Friesian	Argov-Argaman et al. (86)
DGAT1 K232A	Milk production traits+ milk coagulation properties	Italy	Italian Holstein–Friesian	Bobbo et al. (87)
DGAT1 K232A	Milk production traits	Netherlands	Holstein–Friesian	Schennink et al. (7)
DGAT1 K232A	Milk production traits	Netherlands	Holstein–Friesian	Schennink et al. (88)
DGAT1 K232A	Milk production traits	Netherlands	Holstein–Friesian	Duchemin et al. (74)
DGAT1 K232A	Milk fat	Netherlands	Holstein–Friesian	Tzompa-Sosa et al. (65)
DGAT1 K232A	Milk metabolome and proteome	Netherlands	Holstein–Friesian	Lu et al. (37)
DGAT1 K232A	Milk production traits and lactose contents	Netherlands	Holstein–Friesian	Bovenhuis et al. (41)
DGAT1 K232A	Higher milk fat, protein and milk fatty acid	Netherlands	Holstein–Friesian	Bovenhuis et al. (42)
DGAT1 K232A	Higher milk fat, protein and lower milk yield	Netherlands	Holstein–Friesian	van Gastelen et al. (64)
DGAT1 K232A	Milk fatty acids	Netherlands	Holstein–Friesian	van Gastelen et al. (89)
DGAT1 (K232A)	Triacylglycerol in milk fat	Netherlands	Holstein–Friesian	Pacheco-Pappenheim et al. (77)
DGAT1 K232A	Milk production traits	New Zealand	Holstein–Friesian	Grisart et al. (21)
DGAT1 K232A	Milk production traits	Poland	Polish Holstein–Friesian	Vanbergue et al. (63)
DGAT1 K232A	Milk production traits	Poland	Polish Holstein–Friesian	Nowacka-Woszuk et al. (90)
DGAT1 K232A	Milk production traits	Poland	Polish Holstein–Friesian	Szyda et al. (69)
DGAT1 K232A	Milk production traits	Poland	Polish Holstein–Friesian	Komisarek J, Kolenda (75)
DGAT1 K232A	Milk fatty acids	Poland	Polish Holstein–Friesian	Kesek-Wozniak et al. (49)
DGAT1 K232A	Milk fat percentage and fatty acid	Romania	Romanian Holstein	Tăbăran et al. (91)
DGAT1 K232A	Milk production traits	Scotland	Holstein–Friesian	Banos et al. (46)
DGAT1 K232A	Milk production traits	Sweden	Swedish Red and Swedish Holstein	Näslund et al. (47)
DGAT1 K232A	Milk production traits	USA	Holstein–Friesian	Barbosa et al. (92)

### DGAT1 Role in Buffalo Milk Production Traits

Buffalo milk is famous because of its high protein and fat content (82). Consistently, it has been shown that buffalo milk has higher milk fat content than cow milk (93). Thus, it is necessary to characterize DGAT1 in buffalo because of high milk fat yield production than that of cows (94). *DGAT1* gene has been characterized in previously reported studies in buffalo (94–97). Yuan et al. studied *DGAT1* gene and its polymorphisms in Chinese water buffalo. Furthermore, it has been revealed that SNP at exon 17 changes the amino acid sequence Ala residue to Val residue at position 484 in buffalo DGAT1 (95). Consequently, Mishra et al. characterized *DGAT1* gene in six different Indian water buffalo (Murrah, Bhadawari, Tarai, Pandharpuri, Marathwada, and Mehsana). The polymorphisms detected in DGAT1 reported by previous studies were significantly associated with milk production traits and could be a target as genetic markers for improvement of milk fat and milk yield production in water buffalo (95, 96).

Two SNPs (g.11,783 G>A and g.11,785 T>C) at exon 17 of DGAT1 in cattle were also identified in Murrah buffaloes (98). Furthermore, it was documented that SNP-g.11, 785 T>C is associated with milk fat and protein percentages in Brazilian Murrah buffaloes. The two SNPs g.8330T>C and g.9046T>C at exons 17 and 13 in *DGAT1* gene were identified in Riverine, Swamp, and crossbred buffalo. Furthermore, it was revealed that g.9046T>C had been associated with the change in amino acid sequence at position 484 arginine to histidine (99). The SNP g.9046T>C was associated with fat percentage, and buffalo with TT genotype showed a higher association for milk fat percentage than the CC genotype. Similarly, the SNP g.8330T>C of DGAT1 in Riverine buffalo was linked to peak milk yield, total milk yield, and protein percentage. The buffalo having a CC genotype showed a higher ability for milk yield and peak yield and less milk protein than the TT genotype (99). Compared with that of cattle, the high milk fat percentage in buffalo might be due to a fixed K allele (94). In contrast, Silva et al. did not find any effect of SNPs in DGAT1 on milk production traits in buffalo. The reason for this might be due to their distribution on the intron region, which is not essential for amino acid sequences on proteins (100). A recently published study revealed that SNP-8426 C/T at exon 17 of DGAT1 changes the amino acid of Ala to Vla in Iranian buffalo (101). Furthermore, the association of SNP-8426 C/T in DGAT1 with milk production traits might a good choice to consider as a genetic marker for enhancement of milk production in buffalo (101). The DGAT K allele was documented in Indian buffalo and cattle breeds (45). Consistently, the DGAT K232A polymorphism was documented by their association with higher milk fat yield, higher milk fat, and protein percent regulation in buffalo breeds (45, 102). Consistently, c.1053C>T polymorphism in the coding region of exon 17 in DGAT1 was found to be involved with milk production traits in the Italian Mediterranean and Romanian buffalo breeds (103). Furthermore, it was revealed that the T allele might be assumed to be *DGAT1* gene's ancestral state, being found in the majority of the sequenced species. Liu et al. through a GWAS reported the significant effect of DGAT family genes on milk production traits in buffalo (82). The published data show that the DGAT1 role in milk production traits in buffalo is lacking, and further validation is warranted. Besides, we did not find a single study regarding *DGAT1* gene importance in buffalo meat production. So still a huge gap is there to evaluate the association of DGAT1 with buffalo meat production.

### DGAT1 Role in Goat and Sheep Milk Production

Due to the high content of short- and medium-chain fatty acids and smaller fat globules, goat milk is generally considered to have a high nutritional value (104). Goat milk in many countries around the world is of particular economic value and is attractive for many reasons, including its abundance in different nutrients and its health importance (105). Genetic selection programs for dairy goats are still rare in many developing countries, but breeding strategies for the commercial production of milk and meat from goats have been remarkably successful (106). In cheese production, milk fatty acids play an important technical function, while DGAT1 has an essential role in the biosynthesis of TGs exported to the milk. The important role of DGAT1 in the metabolism of milk fat makes *DGAT1* gene an interesting candidate for the genetic variation of milk characteristics in milk goats (107). DGAT1 also plays an essential function in physiological processes that include the synthesis of TAG, such as the absorption of intestinal fat (22, 36), the development of adipose tissue, and mammals' lactation (12, 19).

*DGAT1* gene was characterized in goat by Angiolillo et al.; furthermore, an association study was suggested to explore the relationship of DGAT1 with TAG synthesis in the goat mammary gland (108). It has been documented that miR-145 regulates the fatty acids metabolism in goat mammary epithelial cells. MiR-145 acts on fatty acid regulating genes including DGAT1 in goat mammary epithelial cells. Furthermore, a significant change in fatty acid level was observed by knocking out the MiR-145, inhibiting the DGAT1 expression (109). Consequently, it has been reported that SP1 impairs the milk fatty acid synthesis by decreasing the expression of DGAT1 in goat mammary epithelial cells (110, 111).

#### Polymorphism in Caprine DGAT1 and Their Association With Milk Production

An et al. discovered different polymorphisms (g.407–408insC (in intron 14), g.6852C → T, and g.6798C → T (in exon 7) of *DGAT1* gene in Xinong Saanen (SN) and Guanzhong (GZ) goat breeds. Furthermore, a significant association of DGAT1 in–del (g.407_408insC) with milk yield and fat percentage were observed in Chinese dairy SN and GZ goats. Interestingly, the DGAT1 in–del (g.407_408insC) was not in the coding region but still has a positive effect on milk production traits (2), which might be due to its regulatory effect on the mechanism of mRNA deadenylation and degradation (112). Consistently, four polymorphisms (T21153G, C21154G, A21172C, and A21194T) were identified in DGAT1 of the Iranian Khalkhali goat (113). The variants T21153G and C21154G cause the transformation of serine to glycine amino acids, while A21172C was associated with the change in aspartic acid to alanine amino acid. Furthermore, Evrigh et al. found that four mutations (T21153G, C21154G, A21172C, and A21194T) of DGAT1 in Iranian Khalkhali goat were significantly associated with milk yield and milk protein percentage (113). Martin et al. explored the two SNPs R251L and R396W in goat DGAT1. The SNPs were genotyped in two goat breeds (Saanen and Alpine breeds). The frequencies noted for R251L and R396W mutations in Saanen goat were 3.5 and 13%; however, only R396W was found with a frequency of 7% in the Alpine breed. Moreover, it was found that both the mutations in DGAT1 were associated with a decrease in milk fat; however, the association was not strongly significant for both the SNPs (114). As the two types of mutation lead to substitution in protein sequence (114), further experimental trials are highly warranted for these two SNPs R251L and R396W in DGAT1 with a higher goat population because the sample size was too low in a study by Martine et al. Similarly, Tabaran et al. did not find the significant influence of *DGAT1* gene on milk production traits in sheep and goat. Again, we found the same issue of less number of samples in the experiments of Tabaran et al. (115). For an association study, usually, a large size of population is needed (116). Recently, two important mutations (T703C and T713C) in DGAT1 of the Egyptian Zaraibi goat were identified (117). The polymorphism T713C was found at the coding region associated with the substitution of amino acid isoleucine to threonine. Furthermore, the polymorphism T713C significantly regulated the total solid content of milk, milk yield, and contents in Egyptian Zaraibi goat (117).

#### DGAT1 Role in Sheep Milk Production

The polymorphisms in DGAT1 have been studied for their association with milk production traits in sheep (118–120). Dervishi et al. identified four polymorphisms: two in exon 17 (g.8522C>T and g.8539C>T), one in exon 1 (g.358C>A), and one in intron 10 (g.7457C>A) of DGAT1 in the Spanish Assaf goat breed (121). Furthermore, it was detected that the SNP (g.358C>A) and (g.8522C>T) leads to a change in amino acid sequence at points p.Asp53Glu and p.Arg482Cys. The association study revealed that the sheep having genotype CC showed greater performance in milk fats than the CT genotype. Overall, the four detected SNPs in DGAT1 were associated with milk production traits in the Spanish Assaf goat breed (121). Consistently, in sheep, Scata et al. documented five new mutations in *DGAT1* gene, distributed on exon 17 (g.8539C>T), 5_UTR (g.127C>A), intron 1 (g.1655C>T), intron 2 (g.5553C>T), and intron 10 (g.7492C>T). Additionally, a negative association of polymorphism g.5553C>T with milk fat content in Sarda, Altamurana, and Gentile di Puglia Italian sheep breeds was documented. Consequently, in the Sarda sheep breed, the variant g.127C>A was negatively associated with milk fat content (122).

## DGAT1 Role in Meat Production

DGAT1 is considered a key enzyme that controls the major pathway of TAG synthesis in the adipose tissue (123, 124). In animals, TAG can be found in the liver, small intestine, muscle, and adipose tissue and is considered the main component of intramuscular fat (IMF) and a key role in energy metabolism (125). TAG also facilitates the cell membrane composition and transportation of lipoprotein (126). Furthermore, significant changes in lipid metabolism in several tissues of DGAT1 lacking mice have been documented (127). Consistently, it was validated that overexpression of DGAT1 is associated with raised TG biosynthesis, enhanced fatty acid oxidation, and preserved insulin sensitivity (128). In addition, Ying et al. experimentally proved the influence of DGAT1 TAG synthesis, resistance to insulin, and IMF deposition (129). Furthermore, it was found that DGAT1 overexpression regulated the glucose and lipid metabolism and catalyzed the TAG synthesis reaction, which is responsible for enhancing muscle insulin sensitivity and raising the content of the IMF (129). Furthermore, it was documented that DGAT1 mediates the TAG synthesis and IMF formation, possibly due to its effect on unsaturated fatty acid, insulin, and MAPK signaling (130). Altogether, it was observed that DGAT1 overexpression is associated with lipid and glucose metabolism, thereby facilitating the TAG biosynthesis (130).

### DGAT1 Association With Meat Production Traits in Cattle

Many studies have investigated the effect of *DGAT1* gene on meat production traits in beef cattle (4, 5, 48, 131). The role of *DGAT1* gene for production traits has also been reported in seven cattle breeds (Angus, Belgian Blue, Charolais, Hereford, Holstein–Friesian, Limousin, and Simmental cattle) (132). Consequently, it was documented that *DGAT1* gene may affect the color (48) fat contents of meat in beef cattle (25). Yuan et al. studied the association of two SNPs (c.572A/G and c.1416T/G) in the exon region of *DGAT1* gene with meat production traits in beef cattle. It was documented that SNPs (c.572A/G and c.1416T/G) in DGAT1 have significantly influenced the backfat thickness, longissimus muscle area, marbling score, and fat color in beef cattle (133). The polymorphism DGAT1 K232A is the key variant associated with production including meat production traits. Few reports have illustrated the role of DGAT1 K232A in the regulation of IMF in beef cattle semitendinosus muscle (53, 134–136); however, Ardicli et al. did not find any association of this polymorphism with meat production traits (40). Similarly, a study reported the nonsignificant effect of DGAT1 K232A on various meat traits (percentage of muscle, percentage of fat, and percentage of bones and drip loss) (137). Moreover, Cases et al. did not find any link of DGAT1 K232A polymorphism with meat production traits in Brahman Breed. The frequency of A genotype in DGAT1 in most beef cattle breeds including Charolais (16), Angus (138), Simmental (48), and Russian beef cattle (139) was higher than K genotype. Rump height (RH) in beef cattle has been significantly influenced by DGAT1 K232A polymorphism (140). Consequently, it was explored that DGAT1 K232A is strongly associated with meat fat and tenderness in three Spanish Cattle breeds (Berrenda en Colorado, Berrenda en Negro, and Cardena Andaluza). In addition, higher frequencies of the K allele were documented in all three breeds compared with the A allele (141). The IMF is an important parameter in practice for the evaluation of the nutritional quality of beef; consistently, Wu et al. documented a noteworthy relationship of DGAT1-(10,433 and 10,434)-GC/GC genotype with marbling score and IMF in Chinese Simmental cattle (142). Importantly, the DGAT1 overexpression caused the significant alteration metabolism of fat metabolism, elevated TG synthesis, regulated fatty acid oxidation synthesis, enhanced fatty acid oxidation, and conserved the sensitivity of insulin (128, 143). Based on published literature, we concluded that although DGAT1 has an important role, very limited research studies explored the DGAT1 function of meat production in cattle.

### DGAT1 Role in Sheep and Goat Meat Production

Xu et al. recorded a silent mutation of GCT (Ala487) to GCC (Ala487) on exon 17 of DDAT1 in the Chinese sheep breed. Furthermore, a significant association of the genotypes TT was confirmed with a higher muscle marbling score (*p* <0.05) and IMF content and lower shear force and drip loss rate (144). An SNP (c.69 G>A) on exon 10 of DGAT1 was documented through sequence analysis in Barki, Najdi, and Harri sheep breeds, which causes the substitution of the amino acid (p.Lysine>Arginine). Furthermore, it was revealed that the detected SNP (c.69 G>A) was associated with meat production traits in Barki, Najdi, and Harri breeds (145). Consistently, Armstrong et al. also identified some novel SNPs of DGAT1 in Texel sheep, which were involved in the development of shoulder weight, fat thickness, rib-eye area, and live weights (146). A study detected SNPs on exons 16 and 17 of DGAT1 in the Lohari sheep breed and suggested that these variants might be considered in association research for meat production traits (31). Consequently, Mohammadi et al. reported a significant effect of SNPs on exons 16 and 17 of DGAT1 on fat-tail weight (*p* <0.05) and backfat thickness in the two Iranian sheep breeds (Lori Bakhtiari and Zel). Furthermore, it was illustrated that the CC genotypes of sheep were associated significantly with fat-tail weight and backfat thickness (147). Ala Noshahr and Rafat documented the significant association of polymorphism T487C on exon 17 of the DGAT1 with carcass weight and dressing percentage in Moghani sheep. In addition CC genotypes Moghani breed showed a higher correlation with carcass weight and dressing percentage than TT genotypes (148). The DGAT1 role in goat meat is still not explored. Recently, a copy number variation (CNV) distribution was studied in the goat. It has been documented that the CNV reported in many genes including DGAT1 was associated with muscle development and metabolic processes in the goat (116).

## Conclusions

Based on our review, it has been concluded that DGAT1 can be used as a genetic marker for the improvement of milk production in dairy cattle. In addition, the DGAT1 showed some positive role in the enhancement of meat and carcass fatness quality in beef cattle. However, further studies are warranted to explore the role of DGAT1 in meat production quality improvement in different breeds of cattle. The DGAT1 also regulates the milk production traits in sheep, goat, and buffalo. However, very limited studies are available on DGAT1 association with milk production traits in buffalo, sheep, and goat. *DGAT1* gene showed some influence on meat production variables in sheep and goat, but not a single study reported the association of DGAT1 for meat production in buffalo. Overall, our review documented that DGAT1 has been studied extensively for milk production in milch animals. However, further validation of DGAT1 for their association with milk and meat production improvement is suggested in these animals to fill the gap with further research in this area.

## Author Contributions

MK and ZC: conceptualization and writing—original draft preparation. IK, AK, YL, JM, YM, SL, JX, and ZC: editing and technical review. ZC: visualization and supervision. All authors have read and agreed to the published version of the manuscript.

## Conflict of Interest

The authors declare that the research was conducted in the absence of any commercial or financial relationships that could be construed as a potential conflict of interest.

## Publisher's Note

All claims expressed in this article are solely those of the authors and do not necessarily represent those of their affiliated organizations, or those of the publisher, the editors and the reviewers. Any product that may be evaluated in this article, or claim that may be made by its manufacturer, is not guaranteed or endorsed by the publisher.

## References

[1] Erhardt G Caroli A Rizzi R Luhken G. Milk protein genetic variation and casein haplotype structure in the Original Pinzgauer cattle. J Dairy Sci. (2010) 93:1260–5. 10.3168/jds.2009-2521 20172246

[2] An X Hou J Zhao H Zhu C Yan Q Song Y. Mutations in Caprine DGAT1 and STAT5A genes were associated with milk production traits. Engineering. (2012) 4:30–4. 10.4236/eng.2012.410B008

[3] Månsson HL. Fatty acids in bovine milk fat. Food Nutr Res. (2008) 52:1–3. 10.3402/fnr.v52i0.1821 19109654 PMC2596709

[4] Warner RD Greenwood PL Pethick DW Ferguson DM. Genetic and environmental effects on meat quality. Meat Sci. (2010) 171–83. 10.1016/j.meatsci.2010.04.042 20561754

[5] Li X Ekerljung M Lundström K Lundén A. Association of polymorphisms at DGAT1, leptin, SCD1, CAPN1 and CAST genes with color, marbling and water holding capacity in meat from beef cattle populations in Sweden. Meat Sci. (2013) 94:153–8. 10.1016/j.meatsci.2013.01.010 23501244

[6] Soyeurt H Dardenne P Gillon A Croquet C Vanderick S Mayeres P. Variation in fatty acid contents of milk and milk fat within and across breeds. J Dairy Sci. (2006) 89:4858–65. 10.3168/jds.S0022-0302(06)72534-6 17106116

[7] Schennink A Stoop WM Visker MHPW Heck JML Bovenhuis H Van Der Poel JJ. DGAT1 underlies large genetic variation in milk-fat composition of dairy cows. Anim Genet. (2007) 38:467–73. 10.1111/j.1365-2052.2007.01635.x 17894561

[8] Dekkers JC. Commercial application of marker- and gene-assisted selection in livestock: strategies and lessons. J Anim Sci. (2004) 82:E313–28. 10.2527/2004.8213_supplE313x 15471812

[9] Meredith BK Kearney FJ Finlay EK Bradley DG Fahey AG Berry DP. Genome-wide associations for milk production and somatic cell score in Holstein-Friesian cattle in Ireland. BMC Genet. (2012) 13:21. 10.1186/1471-2156-13-21 22449276 PMC3361482

[10] Narayana SG Schenkel FS Fleming A Koeck A Malchiodi F Jamrozik J. Genetic analysis of groups of mid-infrared predicted fatty acids in milk. J Dairy Sci. (2017) 100:4731–44. 10.3168/jds.2016-12244 28342614

[11] Heimes A Brodhagen J Weikard R Hammon HM Meyerholz MM Petzl W. Characterization of functional traits with focus on udder health in heifers with divergent paternally inherited haplotypes on BTA18. BMC Vet Res. (2019) 15:241. 10.1186/s12917-019-2034-2 31296208 PMC6624885

[12] Spelman RJ Ford CA McElhinney P Gregory GC Snell RG. Characterization of the DGAT1 gene in the New Zealand dairy population. J Dairy Sci. (2002) 85:3514–7. 10.3168/jds.S0022-0302(02)74440-8 12512625

[13] Weller JI Golik M Seroussi E Ezra E Ron M. Population-wide analysis of a QTL affecting milk-fat production in the Israeli Holstein population. J Dairy Sci. (2003) 86:2219–27. 10.3168/jds.S0022-0302(03)73812-0 12836959

[14] Ashwell MS Heyen DW Sonstegard TS Van Tassell CP Da Y VanRaden PM. Detection of quantitative trait loci affecting milk production, health, and reproductive traits in Holstein cattle. J Dairy Sci. (2004) 87:468–75. 10.3168/jds.S0022-0302(04)73186-0 14762090

[15] Nayeri S Sargolzaei M Abo-Ismail MK Miller S Schenkel F Moore SS. Genome-wide association study for lactation persistency, female fertility, longevity, and lifetime profit index traits in Holstein dairy cattle. J Dairy Sci. (2017) 100:1246–58. 10.3168/jds.2016-11770 27889128

[16] Ripoli M V Corva P Giovambattista G. Analysis of a polymorphism in the DGAT1 gene in 14 cattle breeds through PCR-SSCP methods. Res Vet Sci. (2006) 80:287–90. 10.1016/j.rvsc.2005.07.006 16464654

[17] Marete AG Guldbrandtsen B Lund MS Fritz S Sahana G Boichard D. A meta-analysis including pre-selected sequence variants associated with seven traits in three french dairy cattle populations. Front Genet. (2018) 9:522. 10.3389/fgene.2018.00522 30459810 PMC6232291

[18] Lu H Bovenhuis H. Genome-wide association studies for genetic effects that change during lactation in dairy cattle. J Dairy Sci. (2019) 102:7263–76. 10.3168/jds.2018-15994 31155265

[19] Fink T Lopdell TJ Tiplady K Handley R Johnson TJJ Spelman RJ. A new mechanism for a familiar mutation - bovine DGAT1 K232A modulates gene expression through multi-junction exon splice enhancement. BMC Genomics. (2020) 21:591. 10.1186/s12864-020-07004-z 32847516 PMC7449055

[20] Lu H Wang Y Bovenhuis H. Genome-wide association study for genotype by lactation stage interaction of milk production traits in dairy cattle. J Dairy Sci. (2020) 103:5234–45. 10.3168/jds.2019-17257 32229127

[21] Grisart B Coppieters W Farnir F Karim L Ford C Berzi P. Positional candidate cloning of a QTL in dairy cattle: Identification of a missense mutation in the bovine DGAT1 gene with major effect on milk yield and composition. Genome Res. (2002) 12:222–31. 10.1101/gr.224202 11827942

[22] Winter A Krämer W Werner FAO Kollers S Kata S Durstewitz G. Association of a lysine-232/alanine polymorphism in a bovine gene encoding acyl-CoA:Diacylglycerol acyltransferase (DGAT1) with variation at a quantitative trait locus for milk fat content. Proc Natl Acad Sci U S A. (2002) 99:9300–5. 10.1073/pnas.142293799 12077321 PMC123135

[23] Casas E White SN Riley DG Smith TPL Brennemant RA Olson TA. Assessment of single nucleotide polymorphisms in genes residing on chromosomes 14 and 29 for association with carcass composition traits in Bos indicus cattle. J Anim Sci. (2005) 83:13–9. 10.2527/2005.83113x 15583037

[24] Wibowo TA Gaskins CT Newberry RC Thorgaard GH Michal JJ Jiang Z. Genome assembly anchored QTL map of bovine chromosome 14. Int J Biol Sci. (2008) 6:406–14. 10.7150/ijbs.4.406 19043607 PMC2586679

[25] Curi RA Chardulo LAL Arrigoni MDB Silveira AC de Oliveira HN. Associations between LEP, DGAT1 and FABP4 gene polymorphisms and carcass and meat traits in Nelore and crossbred beef cattle. Livest Sci. (2011) 135:244–50. 10.1016/j.livsci.2010.07.013

[26] Urbinati I Stafuzza NB Oliveira MT Chud TCS Higa RH Regitano LC de A. Selection signatures in Canchim beef cattle. J Anim Sci Biotechnol. (2016) 7:29. 10.1186/s40104-016-0089-5 27158491 PMC4858954

[27] Sorbolini S Marras G Gaspa G Dimauro C Cellesi M Valentini A. Detection of selection signatures in Piemontese and Marchigiana cattle, two breeds with similar production aptitudes but different selection histories. Genet Sel Evol. (2015) 47:52. 10.1186/s12711-015-0128-2 26100250 PMC4476081

[28] Amaral MEJ Grant JR Riggs PK Stafuzza NB Filho EAR Goldammer T. A first generation whole genome RH map of the river buffalo with comparison to domestic cattle. BMC Genomics. (2008) 9:631. 10.1186/1471-2164-9-631 19108729 PMC2625372

[29] Devita RJ Pinto S. Current status of the research and development of diacylglycerol o -acyltransferase 1 (DGAT1) inhibitors. J Med Chem. (2013) 56:9820–5. 10.1021/jm4007033 23919406

[30] Muise ES Zhu Y Verras A Karanam BV Gorski J Weingarth D. Identification and characterization of sebaceous gland atrophy-sparing DGAT1 inhibitors. PLoS ONE. (2014) 9:e88908. 10.1371/journal.pone.0088908 24558447 PMC3928314

[31] Nanekarani S Kolivand M Goodarzi M. Polymorphism of a Mutation of DGAT1 Gene in Lori Sheep Breed. J Adv Agric Technol. (2016) 3:38–41. 10.18178/joaat.3.1.38-41

[32] Lehner R Kuksis A Biosynthesis of triacylglycerols. Prog Lipid Res. (1996) 35:169–201. 10.1016/0163-7827(96)00005-7 8944226

[33] Cases S Stone SJ Zhou P Yen E Tow B Lardizabal KD. Cloning of DGAT2, a second mammalian diacylglycerol acyltransferase, and related family members. J Biol Chem. (2001) 276:38870–6. 10.1074/jbc.M106219200 11481335

[34] Farese R V Cases S Smith SJ. Triglyceride synthesis: Insights from the cloning of diacylglycerol acyltransferase. Curr Opin Lipidol. (2000) 11:229–34. 10.1097/00041433-200006000-00002 10882337

[35] Smith SJ Cases S Jensen DR Chen HC Sande E Tow B. Obesity resistance and multiple mechanisms of triglyceride synthesis in mice lacking Dgat. Nat Genet. (2000) 25:87–90. 10.1038/75651 10802663

[36] Cases S Smith SJ Zheng YW Myers HM Lear SR Sande E. Identification of a gene encoding an acyl CoA: diacylglycerol acyltransferase, a key enzyme in triacylglycerol synthesis. Proc Natl Acad Sci USA. (1998) 95:13018–23. 10.1073/pnas.95.22.13018 9789033 PMC23692

[37] Lu J Boeren S van Hooijdonk T Vervoort J Hettinga K. Effect of the DGAT1 K232A genotype of dairy cows on the milk metabolome and proteome. J Dairy Sci. (2015) 98:3460–9. 10.3168/jds.2014-8872 25771043

[38] Molee A Duanghaklang N Na-Lampang P. Effects of Acyl-CoA: Diacylglycerol acyl transferase 1 (DGAT1) gene on milk production traits in crossbred Holstein dairy cattle. Trop Anim Health Prod. (2012) 44:751–5. 10.1007/s11250-011-9959-1 21881942

[39] Fontanesi L Calò DG Galimberti G Negrini R Marino R Nardone A. A candidate gene association study for nine economically important traits in Italian Holstein cattle. Anim Genet. (2014) 45:576–80. 10.1111/age.12164 24796806

[40] Ardicli S Soyudal B Samli H Dincel D Balci F. Effect of STAT1, OLR1, CSN1S1, CSN1S2, and DGAT1 genes on milk yield and composition traits of Holstein breed. Rev Bras Zootec. (2018) 47:47. 10.1590/rbz4720170247

[41] Bovenhuis H Visker MHPW Poulsen NA Sehested J van Valenberg HJF van Arendonk JAM. Effects of the diacylglycerol o-acyltransferase 1 (DGAT1) K232A polymorphism on fatty acid, protein, and mineral composition of dairy cattle milk. J Dairy Sci. (2016) 99:3113–23. 10.3168/jds.2015-10462 26898284

[42] Bovenhuis H Visker MHPW van Valenberg HJF Buitenhuis AJ van Arendonk JAM. Effects of the DGAT1 polymorphism on test-day milk production traits throughout lactation. J Dairy Sci. (2015) 98:6572–82. 10.3168/jds.2015-9564 26142855

[43] Bouwman AC Bovenhuis H Visker MHPW van Arendonk JAM. Genome-wide association of milk fatty acids in Dutch dairy cattle. BMC Genet. (2011) 12:43. 10.1186/1471-2156-12-43 21569316 PMC3120725

[44] Jiang J Ma L Prakapenka D VanRaden PM Cole JB Da Y. A large-scale genome-wide association study in U.S. Holstein cattle. Front Genet. (2019) 10. 10.3389/fgene.2019.00412 31139206 PMC6527781

[45] Tantia MS Vijh RK Mishra BP Mishra B Kumar STB Sodhi M. DGAT1 and ABCG2 polymorphism in Indian cattle (Bos indicus) and buffalo (Bubalus bubalis) breeds. BMC Vet Res. (2006) 2:32. 10.1186/1746-6148-2-32 17087837 PMC1636029

[46] Banos G Woolliams JA Woodward BW Forbes AB Coffey MP. Impact of single nucleotide polymorphisms in leptin, leptin receptor, growth hormone receptor, and diacylglycerol acyltransferase (DGAT1) gene loci on milk production, feed, and body energy traits of UK dairy cows. J Dairy Sci. (2008) 91:3190–200. 10.3168/jds.2007-0930 18650297

[47] Näslund J Fikse WF Pielberg GR Lundén A. Frequency and effect of the bovine Acyl-CoA:Diacylglycerol acyltransferase 1 (DGAT1) K232A polymorphism in Swedish dairy cattle. J Dairy Sci. (2008) 91:2127–34. 10.3168/jds.2007-0330 18420644

[48] Ardicli S Samli H Dincel D Ekiz B Yalcintan H Vatansever B. Relationship of the bovine IGF1, TG, DGAT1 and MYF5 genes to meat colour, tenderness and cooking loss. J Hell Vet Med Soc. (2018) 69:1077–87. 10.12681/jhvms.18879

[49] Kesek-Wozniak MM Wojtas E Zielak-Steciwko AE. Impact of SNPs in ACACA, SCD1, and DGAT1 genes on fatty acid profile in bovine milk with regard to lactation phases. Animals. (2020) 10:1–13. 10.3390/ani10060997 32521715 PMC7341249

[50] Lehnert K Ward H Berry SD Ankersmit-Udy A Burrett A Beattie EM. Phenotypic population screen identifies a new mutation in bovine DGAT1 responsible for unsaturated milk fat. Sci Rep. (2015) 5:8484. 10.1038/srep08484 25719731 PMC4341421

[51] Grisart B Farnir F Karim L Cambisano N Kim JJ Kvasz A. Genetic and functional confirmation of the causality of the DGAT1 K232A quantitative trait nucleotide in affecting milk yield and composition. Proc Natl Acad Sci U S A. (2004) 101:2398–403. 10.1073/pnas.0308518100 14983021 PMC356962

[52] Demeter MR Schopen G Oude Lansink A Meuwissen M. van A. Effects of milk fat composition, DGAT1, and SCD1 on fertility traits in Dutch Holstein cattle. J Dairy Sci. (2009) 92:5720–9. 10.3168/jds.2009-2069 19841232

[53] Thaller G Kühn C Winter A Ewald G Bellmann O Wegner J. DGAT1, a new positional and functional candidate gene for intramuscular fat deposition in cattle. Anim Genet. (2003) 34:354–7. 10.1046/j.1365-2052.2003.01011.x 14510671

[54] Sanders K Bennewitz J Reinsch N Thaller G Prinzenberg EM Kühn C. Characterization of the DGAT1 mutations and the CSN1S1 promoter in the German Angeln dairy cattle population. J Dairy Sci. (2006) 89:3164–74. 10.3168/jds.S0022-0302(06)72590-5 16840633

[55] Pareek CS Czarnik U Zabolewicz T Pareek RS Walawski K. DGAT1 K232A quantitative trait nucleotide polymorphism in Polish Black-and-White cattle. J Appl Genet. (2005) 46:85–7. 10.1107/S0907444998010464 15741668

[56] Strzałkowska N Siadkowska E Słoniewski K Krzyzewski J Zwierzchowski L. Effect of the DGAT1 gene polymorphism on milk production traits in Black-and-White (Friesian) cows. Anim Sci Pap Reports. (2005) 23:189–97. 10.1556/AVet.56.2008.2.5 18669245

[57] Sun D Jia J Ma Y Wang Y Yu Y Zhang Y. Effects of DGAT1 and GHR on milk yield and milk composition in the Chinese dairy population. Anim Genet. (2009) 40:997–1000. 10.1111/j.1365-2052.2009.01945.x 19781040

[58] Khan MZ Wang D Liu L Usman T Wen H Zhang R. Significant genetic effects of JAK2 and DGAT1 mutations on milk fat content and mastitis resistance in Holsteins. J Dairy Res. (2019) 86:388–93. 10.1017/S0022029919000682 31779717

[59] Li F Cai C Qu K Liu J Jia Y Hanif Q. DGAT1 K232A polymorphism is associated with milk production traits in Chinese cattle. Anim Biotechnol. (2020) 15:1–5. 10.1080/10495398.2020.1711769 32053037

[60] Gautier M Capitan A Fritz S Eggen A Boichard D Druet T. Characterization of the DGAT1 K232A and variable number of tandem repeat polymorphisms in French dairy cattle. J Dairy Sci. (2007) 90:2980–8. 10.3168/jds.2006-707 17517739

[61] Berry DP Howard D O'Boyle P Waters S Kearney JF McCabe M. Associations between the K232A polymorphism in the diacylglycerol-O-transferase 1 (DGAT1) gene and performance in Irish Holstein-Friesian dairy cattle. Irish J Agric Food Res. (2010) 49:1–9. 10.2307/20788561

[62] Oliveira HR Lourenco DAL Masuda Y Misztal I Tsuruta S Jamrozik J. Single-step genome-wide association for longitudinal traits of Canadian Ayrshire, Holstein, and Jersey dairy cattle. J Dairy Sci. (2019) 102:9995–1011. 10.3168/jds.2019-16821 31477296

[63] Vanbergue E Peyraud JL Guinard-Flament J Charton C Barbey S Lefebvre R. Effects of DGAT1 K232A polymorphism and milking frequency on milk composition and spontaneous lipolysis in dairy cows. J Dairy Sci. (2016) 99:5739–49. 10.3168/jds.2015-10731 27132096

[64] van Gastelen S Visker MHPW Edwards JE Antunes-Fernandes EC Hettinga KA Alferink SJJ. Linseed oil and DGAT1 K232A polymorphism: Effects on methane emission, energy and nitrogen metabolism, lactation performance, ruminal fermentation, and rumen microbial composition of Holstein-Friesian cows. J Dairy Sci. (2017) 100:8939–57. 10.3168/jds.2016-12367 28918153

[65] Tzompa-Sosa DA van Valenberg HJF van Aken GA Bovenhuis H. Milk fat triacylglycerols and their relations with milk fatty acid composition, DGAT1 K232A polymorphism, and milk production traits. J Dairy Sci. (2016) 99:3624–31. 10.3168/jds.2015-10592 26971154

[66] Mach N Blum Y Bannink A Causeur D Houee-Bigot M Lagarrigue S. Pleiotropic effects of polymorphism of the gene diacylglycerol-O-transferase 1 (DGAT1) in the mammary gland tissue of dairy cows. J Dairy Sci. (2012) 95:4989–5000. 10.3168/jds.2012-5348 22916903

[67] Suchoki T Komisarek J Szyda J. Testing candidte gene affects on milk production traits in dairy cattle under various parameterizations and modes of inheritance. J Dairy Sci. (2010) 93:2703–17. 10.3168/jds.2009-2550 20494180

[68] Strucken EM de Koning DJ Rahmatalla SA Brockmann GA. Lactation curve models for estimating gene effects over a timeline. J Dairy Sci. (2011) 94:442–9. 10.3168/jds.2009-2932 21183055

[69] Szyda J Morek-Kopeć M Komisarek J Zarnecki A. Evaluating markers in selected genes for association with functional longevity of dairy cattle. BMC Genet. (2011) 12. 10.1186/1471-2156-12-30 21392379 PMC3061949

[70] Bionaz M Periasamy K Rodriguez-Zas SL Hurley WL Loor JJ. A novel dynamic impact approach (DIA) for functional analysis of time-course omics studies: Validation using the bovine mammary transcriptome. PLoS ONE. (2012) 7. 10.1371/journal.pone.0032455 22438877 PMC3306320

[71] Bionaz M Loor JJ. Gene networks driving bovine milk fat synthesis during the lactation cycle. BMC Genomics. (2008) 9. 10.1186/1471-2164-9-366 18671863 PMC2547860

[72] Wickramasinghe S Rincon G Islas-Trejo A Medrano JF. Transcriptional profiling of bovine milk using RNA sequencing. BMC Genomics. (2012) 13:45. 10.1186/1471-2164-13-45 22276848 PMC3285075

[73] Gao Y Lin X Shi K Yan Z Wang Z. Bovine Mammary Gene Expression Profiling during the Onset of Lactation. PLoS ONE. (2013) 8:e70393. 10.1371/journal.pone.0070393 23990904 PMC3749150

[74] Duchemin S Bovenhuis H Stoop WM Bouwman AC van Arendonk JAM Visker MHPW. Genetic correlation between composition of bovine milk fat in winter and summer, and DGAT1 and SCD1 by season interactions. J Dairy Sci. (2013) 96:592–604. 10.3168/jds.2012-5454 23127906

[75] Komisarek J Kolenda M. The effect of DGAT1 polymorphism on milk production traits in dairy cows depending on environmental temperature. Turkish J Vet Anim Sci. (2016) 40:251–4. 10.3906/vet-1508-7

[76] Shahzad K Akbar H Vailati-Riboni M Basiricò L Morera P Rodriguez-Zas SL. The effect of calving in the summer on the hepatic transcriptome of Holstein cows during the peripartal period. J Dairy Sci. (2015) 98:5401–13. 10.3168/jds.2015-9409 26074246

[77] Pacheco-Pappenheim S Yener S van Valenberg HJF Tzompa-Sosa DA Bovenhuis H. The DGAT1 K232A polymorphism and feeding modify milk fat triacylglycerol composition. J Dairy Sci. (2019) 102:6842–52. 10.3168/jds.2019-16554 31178185

[78] Fürbass R Winter A Fries R Kühn C. Alleles of the bovine DGAT1 variable number of tandem repeat associated with a milk fat QTL at chromosome 14 can stimulate gene expression. Physiol Genomics. (2006) 25:116–20. 10.1152/physiolgenomics.00145.2005 16534144

[79] Houaga I Muigai AWT Ng'ang'a FM Ibeagha-Awemu EM Kyallo M Youssao IAK. Milk fatty acid variability and association with polymorphisms in SCD1 and DGAT1 genes in White Fulani and Borgou cattle breeds. Mol Biol Rep. (2018) 45:1849–62. 10.1007/s11033-018-4331-4 30168097 PMC6267235

[80] Lacorte GA Machado MA Martinez ML Campos AL Maciel RP Verneque RS. DGAT1 K232A polymorphism in Brazilian cattle breeds. Genet Mol Res. (2006) 5:475–82. 10.1590/S1415-47572006000200033 17117362

[81] Carvajal AM Huircan P Dezamour JM Subiabre I Kerr B Morales R. Milk fatty acid profile is modulated by DGAT1 and SCD1 genotypes in dairy cattle on pasture and strategic supplementation. Genet Mol Res. (2016) 15:7057. 10.4238/gmr.15027057 27173340

[82] Liu J Wang Z Li J Li H Yang L. Genome-wide identification of Diacylglycerol Acyltransferases (DGAT) family genes influencing Milk production in Buffalo. BMC Genet. (2020) 21:1–14. 10.1186/s12863-020-0832-y 32138658 PMC7059399

[83] Kadlecova V Nemeckova D Jecminkova K Stadnik L. The effects of polymorphism in the DGAT1 gene on energy balance and milk production traits in primiparous holstein cows during the first six months of lactation. Bulg J Agric Sci. (2014) 20:203–9. Available online at: http://agrojournal.org/20/01-34

[84] Kühn C Thaller G Winter A Bininda-Emonds ORP Kaupe B Erhardt G. Evidence for multiple alleles at the DGAT1 locus better explains a quantitative trait locus with major effect on milk fat content in cattle. Genet. (2004) 167:1873–81. 10.1534/genetics.103.022749 15342525 PMC1470998

[85] Kuehn C Edel C Weikard R Thaller G. Dominance and parent-of-origin effects of coding and non-coding alleles at the acylCoA-diacylglycerol-acyltransferase (DGAT1) gene on milk production traits in German Holstein cows. BMC Genet. (2007) 8. 10.1186/1471-2156-8-62 17892573 PMC2129099

[86] Argov-Argaman N Mida K Cohen BC Visker M Hettinga K. Milk Fat Content and DGAT1 Genotype Determine Lipid Composition of the Milk Fat Globule Membrane. PLoS ONE. (2013) 8:e68707. 10.1371/journal.pone.0068707 23874734 PMC3715532

[87] Bobbo T Tiezzi F Penasa M De Marchi M Cassandro M. Short communication: Association analysis of diacylglycerol acyltransferase (DGAT1) mutation on chromosome 14 for milk yield and composition traits, somatic cell score, and coagulation properties in Holstein bulls. J Dairy Sci. (2018) 101:8087–91. 10.3168/jds.2018-14533 30007808

[88] Schennink A Bovenhuis H Léon-Kloosterziel KM Van Arendonk JAM Visker MHPW. Effect of polymorphisms in the FASN, OLR1, PPARGC1A, PRL and STAT5A genes on bovine milk-fat composition. Anim Genet. (2009) 40:909–16. 10.1111/j.1365-2052.2009.01940.x 19719788

[89] van Gastelen S Antunes-Fernandes EC Hettinga KA Dijkstra J. (2018). Short communication: The effect of linseed oil and DGAT1 K232A polymorphism on the methane emission prediction potential of milk fatty acids. J Dairy Sci. (2018) 101:1–6. 10.3168/jds.2017-14131 29550127

[90] Nowacka-Woszuk J Noskowiak A Strabel T Jankowski T Switoński M. An effect of the DGAT1 gene polymorphism on breeding value of Polish Holstein-Friesian sires. Anim Sci Pap Reports. (2008) 26:17–23. 10.1016/j.anifeedsci.2007.01.017

[91] Tăbăran A Balteanu VA Gal E Pusta D Mihaiu R Dan SD. Influence of DGAT1 K232A polymorphism on milk fat percentage and fatty acid profiles in romanian holstein cattle. Anim Biotechnol. (2015) 26:105–11. 10.1080/10495398.2014.933740 25380462

[92] Barbosa DS M,V Sonstegars M Thallman T Connor R Schnabel E Van Tassell R Characterization of DGAT1 allelic effects in a sample of North American Holstein cattle. Anim biotechnol. (2010) 21:88–99. 10.1080/10495390903504625 20379885

[93] Khedkar CD Kalyankar SD Deosarkar SS. Buffalo milk. Encyclopedia of Food and Health. (2016) 84:522–8. 10.1016/B978-0-12-384947-2.00093-3

[94] Özdil F Ilhan F. DGAT1-exon8 polymorphism in Anatolian buffalo. Livest Sci. (2012) 149:83–7. 10.1016/j.livsci.2012.06.030

[95] Yuan J Zhou J Deng X Hu X Li N. Molecular cloning and single nucleotide polymorphism detection of buffalo DGAT1 gene. Biochem Genet. (2007) 45:611–21. 10.1007/s10528-007-9100-3 17592768

[96] Mishra B Tantia MS Kumar STB Vijh RK. Characterization of the DGAT1 gene in the Indian buffalo (Bubalus bubalis). Genet Mol Biol. (2007) 30:1097–100. 10.1590/S1415-47572007000600012

[97] Venkatachalapathy RT Sharma A Sukla S Bhattacharya TK. Cloning and characterization of DGAT1 gene of Riverine buffalo. DNA Seq - J DNA Seq Mapp. (2008) 19:177–84. 10.1080/10425170701461748 18464039

[98] de Freitas AC de Camargo GMF Stafuzza NB Aspilcueta-Borquis RR Venturini GC Dias MM. Genetic association between SNPs in the DGAT1 gene and milk production traits in Murrah buffaloes. Trop Anim Health Prod. (2016) 48:1421–6. 10.1007/s11250-016-1110-x 27469895

[99] Li J Liu S Li Z Zhang S Hua G Salzano A. DGAT1 polymorphism in Riverine buffalo, Swamp buffalo and crossbred buffalo. J Dairy Res. (2018) 85:412–5. 10.1017/S0022029918000468 30070182

[100] Silva CS Silva Filho E Matos AS Schierholt AS Costa MR Marques LC. Polymorphisms in the DGAT1 gene in buffaloes (Bubalus bubalis) in the amazon. Genet Mol Res. (2016) 15:8720. 10.4238/gmr.15038720 27706739

[101] Naserkheil M Miraie-Ashtiani SR Sadeghi M Nejati-Javaremi A Park CW Min KS. Exploring novel single nucleotide polymorphisms and haplotypes of the diacylglycerol O-acyltransferase 1 (DGAT1) gene and their effects on protein structure in Iranian buffalo. Genes and Genomics. (2019) 41:1265–71. 10.1007/s13258-019-00854-2 31388977

[102] Shi DS Wang J Yang Y Lu FH Li XP Liu QY. DGAT1, GH, GHR, PRL and PRLR Polymorphism in Water Buffalo (Bubalus bubalis). Reprod Domest Anim. (2012) 47:328–34. 10.1111/j.1439-0531.2011.01876.x 21883511

[103] Gu M Cosenza G Nicolae I Bota A Guo Y Di Stasio L. Transcript analysis at DGAT1 reveals different mRNA profiles in river buffaloes with extreme phenotypes for milk fat. J Dairy Sci. (2017) 100:8265–76. 10.3168/jds.2017-12771 28780112

[104] Haenlein GFW. Goat milk in human nutrition. Small Rumin Res. (2004) 51:155–63. 10.1016/j.smallrumres.2003.08.010

[105] Chen D Li XY Zhao X Qin YS Zhang XX Li J. Proteomics and microstructure profiling of goat milk protein after homogenization. J Dairy Sci. (2019) 102:3839–50. 10.3168/jds.2018-15363 30827554

[106] Vacca GM Stocco G Dettori ML Summer A Cipolat-Gotet C Bittante G. Cheese yield, cheesemaking efficiency, and daily production of 6 breeds of goats. J Dairy Sci. (2018) 101:7817–32. 10.3168/jds.2018-14450 30126595

[107] Dixit SP Sivalingam J Tyagi AK Saroha V Sharma A Nagda RK. Association of novel SNPs in the candidate genes affecting caprine milk fatty acids related to human health. Meta Gene. (2015) 4:45–56. 10.1016/j.mgene.2015.01.004 25853060 PMC4372655

[108] Angiolillo A Amills M Urrutia B Doménech A Sastre Y Badaoui B. Identification of a single nucleotide polymorphism at intron 16 of the caprine Acyl-Coenzyme A: Diacylglycerol acyltransferase 1 (DGAT1) gene. J Dairy Res. (2007) 74:47–51. 10.1017/S0022029906002196 16978454

[109] Huang L Tian H Luo J Song N Zhang T Wu J. / Cas9 based knockout of miR-145 affects intracellular fatty acid metabolism by targeting INSIG1 in goat mammary epithelial cells. J Agric Food Chem. (2020) 68:5138–46. 10.1021/acs.jafc.0c00845 32299216

[110] Zhu J Sun Y Luo J Wu M Li J Cao Y. Specificity protein 1 regulates gene expression related to fatty acid metabolism in goat mammary epithelial cells. Int J Mol Sci. (2015) 16:1806–20. 10.3390/ijms16011806 25594872 PMC4307335

[111] Zhu JJ Luo J Xu HF Wang H Loor JJ. Short communication: Altered expression of specificity protein 1 impairs milk fat synthesis in goat mammary epithelial cells. J Dairy Sci. (2016) 99:4893–8. 10.3168/jds.2015-10733 26995134

[112] Clement JQ Maiti S Wilkinson MF. Localization and stability of introns spliced from the pem homeobox gene. J Biol Chem. (2001) 276:16919–30. 10.1074/jbc.M005104200 11278282

[113] Evrigh NH Nourouzi Z Vahedi V Benemar HA. Genetic association between the variation of DGAT1 gene and milk production traits in Khalkhali goat. Agric Food. (2018) 6:188–94. 10.5713/ajas.2006.315

[114] Martin P Palhière I Maroteau C Bardou P Canale-Tabet K Sarry J. A genome scan for milk production traits in dairy goats reveals two new mutations in Dgat1 reducing milk fat content. Sci Rep. (2017) 7:1872. 10.1038/s41598-017-02052-0 28500343 PMC5431851

[115] Tabaran A Mihaiu M Dan SD Reget O Pivariu B Cordis I. Identification of Polymorphism in Goat and Sheep DGAT1 Gene Associated with Milk Production Traits. Bull Univ Agric Sci Vet Med Cluj-Napoca Vet Med. (2014) 71:283–6. 10.15835/buasvmcn-vm:9555

[116] Liu M Zhou Y Rosen BD Van Tassell CP Stella A Tosser-Klopp G. Diversity of copy number variation in the worldwide goat population. Heredity. (2019) 122:636–46. 10.1038/s41437-018-0150-6 30401973 PMC6462038

[117] Eid JI Teleb DF Mohamed SA El-Ghor AA. DGAT1 polymorphism in Egyptian Zaraibi goat breed and their association with milk yield and composition. J Basic Appl Zool. (2020) 81:1–7. 10.1186/s41936-020-00176-w

[118] García-Fernández M Gutiérrez-Gil B Sánchez JP Morán JA García-Gámez E Álvarez L. The role of bovine causal genes underlying dairy traits in Spanish Churra sheep. Anim Genet. (2011) 42:415–20. 10.1111/j.1365-2052.2010.02162.x 21749424

[119] Ozmen O Kul S. Polymorphism of goat DGAT1 gene and their association with milk production traits. Indian J Anim Sci. (2014) 84:867–71. 10.1021/jf505199a 25552290

[120] Gutiérrez-Gil B Arranz JJ Pong-Wong R García-Gámez E Kijas J Wiener P. Application of selection mapping to identify genomic regions associated with dairy production in sheep. PLoS ONE. (2014) 9:1. 10.1371/journal.pone.0094623 24788864 PMC4006912

[121] Dervishi E Serrano M Joy M Sarto P Somera A González-Calvo L. Structural characterisation of the acyl CoA: Diacylglycerol acyltransferase 1 (DGAT1) gene and association studies with milk traits in Assaf sheep breed. Small Rumin Res. (2015) 131:78–84. 10.1016/j.smallrumres.2015.08.015

[122] Scatà MC Napolitano F Casu S Carta A De Matteis G Signorelli F. Ovine acyl CoA:diacylglycerol acyltransferase 1- molecular characterization, polymorphisms and association with milk traits. Anim Genet. (2009) 40:737–42. 10.1111/j.1365-2052.2009.01909.x 19466941

[123] Yu YH Ginsberg HN. The role of acyl-CoA:diacylglycerol acyltransferase (DGAT) in energy metabolism. Ann Med. (2004) 36:252–61. 10.1080/07853890410028429 15224651

[124] Yen CLE Nelson DW Yen MI. Intestinal triacylglycerol synthesis in fat absorption and systemic energy metabolism. J Lipid Res. (2015) 56:489–501. 10.1194/jlr.R052902 25231105 PMC4340298

[125] Listrat A Lebret B Louveau I Astruc T Bonnet M Lefaucheur L. How muscle structure and composition influence meat and flesh quality. Sci. World J. (2016) 3182746. 10.1155/2016/3182746 27022618 PMC4789028

[126] Coleman RA Mashek DG. Mammalian triacylglycerol metabolism: Synthesis, lipolysis, and signaling. Chem. Rev. (2011) 6359–86. 10.1021/cr100404w 21627334 PMC3181269

[127] Chen H Smith S Ladha Z. Increased insulin and leptin sensitivity in mice lacking acyl CoA: diacylglycerol acyltransferase 1. J Clin Investig. (2002) 109:1049–55. 10.1172/JCI0214672 11956242 PMC150948

[128] Timmers S de Vogel-van den Bosch J Hesselink MKC van Beurden D Schaart G Ferraz MJ. Paradoxical increase in TAG and DAG content parallel the insulin sensitizing effect of unilateral DGAT1 overexpression in rat skeletal muscle. PLoS ONE. (2011) 6:e14503. 10.1371/journal.pone.0014503 21264296 PMC3021516

[129] Ying F Gu H Xiong Y Zuo B. Analysis of Differentially Expressed Genes in Gastrocnemius Muscle between DGAT1 Transgenic Mice and Wild-Type Mice. Biomed Res Int. (2017) 2017:1–9. 10.1155/2017/5404682 28386555 PMC5366756

[130] Fei X Yu J Li Y. Deng X.CrMAPK3 regulates the expression of iron-dificiency-responsive genes in chlamydomonas reinhardtii. BMC Biochem. (2017) 18:6. 10.1186/s12858-017-0081-5 28511672 PMC5434638

[131] Collis E Fortes MRS Zhang Y Tier B Schutt K Barendse W. Genetic variants affecting meat and milk production traits appear to have effects on reproduction traits in cattle. Anim Genet. (2012) 43:442–6. 10.1111/j.1365-2052.2011.02272.x 22497268

[132] Zhao F McParland S Kearney F Du L Berry DP. Detection of selection signatures in dairy and beef cattle using high-density genomic information. Genet Sel Evol. (2015) 47:49. 10.1186/s12711-015-0127-3 26089079 PMC4472243

[133] Yuan Z Li J Li J Gao X Gao H Xu S. Effects of DGAT1 gene on meat and carcass fatness quality in Chinese commercial cattle. Mol Biol Rep. (2013) 40:1947–54. 10.1007/s11033-012-2251-2 23143182

[134] Pannier L Mullen AM Hamill RM Stapleton PC Sweeney T. Association analysis of single nucleotide polymorphisms in DGAT1, TG and FABP4 genes and intramuscular fat in crossbred Bos taurus cattle. Meat Sci. (2010) 85:515–8. 10.1016/j.meatsci.2010.02.025 20416823

[135] Tait RG Shackelford SD Wheeler TL King DA Keele JW Casas E. CAPN1, CAST, and DGAT1 genetic effects on preweaning performance, carcass quality traits, and residual variance of tenderness in a beef cattle population selected for haplotype and allele equalization. J Anim Sci. (2014) 92:5382–93. 10.2527/jas.2014-8211 25414103

[136] Moore SS Li C Basarab J Snelling WM Kneeland J Murdoch B. Fine mapping of quantitative trait loci and assessment of positional candidate genes for backfat on bovine chromosome 14 in a commercial line of Bos taurus. J Anim Sci. (2003) 81:1919–25. 10.2527/2003.8181919x 12926773

[137] Anna T Klára V Michal G Martina M Radovan K Nina M. The impact of diacylglycerol O-acyltransferase 1 gene polymorphism on carcass traits in cattle. J Cent Eur Agric. (2019) 20:12–18. 10.5513/JCEA01/20.1.2411

[138] Kaupe B Brandt H Prinzenberg EM Erhardt G. Joint analysis of the influence of CYP11B1 and DGAT1 genetic variation on milk production, somatic cell score, conformation, reproduction, and productive lifespan in German Holstein cattle. J Anim Sci. (2007) 85:11–21. 10.2527/jas.2005-753 17179535

[139] Gorlov IF Fedunin AA Randelin DA Sulimova GE. Polymorphisms of bGH, RORC, and DGAT1 genes in Russian beef cattle breeds. Russ J Genet. (2014) 50:1302–7. 10.1134/S1022795414120035 25975152

[140] Souza FRP Mercadante MEZ Fonseca LFS Ferreira LMS Regatieri IC Ayres DR. Assessment of DGAT1 and LEP gene polymorphisms in three Nelore (Bos indicus) lines selected for growth and their relationship with growth and carcass traits. J Anim Sci. (2010) 88:435–41. 10.2527/jas.2009-2174 19820053

[141] Rodero E González A Avilés C Luque M. Conservation of endangered spanish cattle breeds using markers of candidate genes for meat quality. Anim Biotechnol. (2013) 24:15–24. 10.1080/10495398.2012.737394 23394366

[142] Wu XX Yang ZP Shi XK Li JY Ji DJ Mao YJ. Association of SCD1 and DGAT1 SNPs with the intramuscular fat traits in Chinese Simmental cattle and their distribution in eight Chinese cattle breeds. Mol Biol Rep. (2012) 39:1065–71. 10.1007/s11033-011-0832-0 21607624

[143] Liu L Shi X Cheol SC Shulman GI Klaus K Nair KS. Paradoxical coupling of triglyceride synthesis and fatty acid oxidation in skeletal muscle overexpressing DGAT1. Diabetes. (2009) 58:2516–24. 10.2337/db08-1096 19675136 PMC2768165

[144] Xu QL Chen YL Ma RX Xue P. Polymorphism of DGAT1 associated with intramuscular fat-mediated tenderness in sheep. J Sci Food Agric. (2009) 89:232–7. 10.1002/jsfa.3431

[145] Altwaty NH Salem LM Mahrous KF. Single nucleotide polymorphisms in the growth hormone receptor gene and Alu1 polymorphisms in the diacylglycerol acyltransferase 1 gene as related to meat production in sheep. Vet World. (2020) 13:884–9. 10.14202/vetworld.2020.884-889 32636583 PMC7311874

[146] Armstrong E Ciappesoni G Iriarte W Da Silva C Macedo F Navajas EA. Novel genetic polymorphisms associated with carcass traits in grazing Texel sheep. Meat Sci. (2018) 145:202–8. 10.1016/j.meatsci.2018.06.014 29982074

[147] Mohammadi H Shahrebabak MM Sadeghi M. Association Between Single Nucleotide Polymorphism in the Ovine DGAT1 Gene and Carcass Traits in Two Iranian Sheep Breeds. Anim Biotechnol. (2013) 24:159–67. 10.1080/10495398.2013.763816 23777346

[148] Ala Noshahr FA Rafat SA. Polymorphism of DGAT1 gene and its relationship with carcass weight and polymorphism of DGAT1 gene and its rela ti onship with carcass weight and dressing percentage in moghani sheep breed. Iran J Applied Anim Sci. (2014) 4:331–4. 10.18178/JOAAT.3.1.38-41

